# Differential Susceptibility and Innate Immune Response of *Aedes aegypti* and *Aedes albopictus* to the Haitian Strain of the Mayaro Virus

**DOI:** 10.3390/v11100924

**Published:** 2019-10-09

**Authors:** Fodé Diop, Haoues Alout, Cheikh Tidiane Diagne, Michèle Bengue, Cécile Baronti, Rodolphe Hamel, Loïc Talignani, Florian Liegeois, Julien Pompon, Ronald E Morales Vargas, Antoine Nougairède, Dorothée Missé

**Affiliations:** 1 MIVEGEC-IRD, Univ. Montpellier, CNRS, 34394 Montpellier, France; fodediop@hotmail.com (F.D.); c.diagne@icloud.com (C.T.D.); michele.bengue@ird.fr (M.B.); rodolphe.hamel@ird.fr (R.H.); loic.talignani@ird.fr (L.T.); florian.liegeois@ird.fr (F.L.); julien.pompon@ird.fr (J.P.); 2 ASTRE, INRA CIRAD (UMR117), 34394 Montpellier, France; haoues.alout@cirad.fr; 3 Unité des virus émergents, Aix Marseille Univ-IRD 190, Inserm 1207-IHU Méditerranée Infection, 13385 Marseille, France; cecile.baronti@univ-amu.fr (C.B.); antoine.nougairede@univ-amu.fr (A.N.); 4 Department of Medical Entomology, Faculty of Tropical Medicine, Mahidol University, Bangkok 10400, Thailand; ronald.mor@mahidol.ac.th

**Keywords:** arbovirus, Mayaro virus, *Aedes aegypti*, *Aedes albopictus*, chondrocytes, ML26A, Niemann–Pick type C1

## Abstract

Mayaro (MAYV) is an emerging arthropod-borne virus belonging to the Alphavirus genus of the *Togaviridae* family. Although forest-dwelling *Haemagogus* mosquitoes have been considered as its main vector, the virus has also been detected in circulating *Aedes ssp* mosquitoes. Here we assess the susceptibility of *Aedes aegypti* and *Aedes albopictus* to infection with MAYV and their innate immune response at an early stage of infection. *Aedes albopictus* was more susceptible to infection with MAYV than *Ae. aegypti.* Analysis of transcript levels of twenty immunity-related genes by real-time PCR in the midgut of both mosquitoes infected with MAYV revealed increased expression of several immune genes, including CLIP-domain serine proteases, the anti-microbial peptides defensin A, E, cecropin E, and the virus inducible gene. The regulation of certain genes appeared to be *Aedes* species-dependent. Infection of *Ae. aegypti* with MAYV resulted in increased levels of myeloid differentiation2-related lipid recognition protein (*ML26A*) transcripts, as compared to *Ae. albopictus*. Increased expression levels of thio-ester-containing protein 22 (*TEP22*) and Niemann–Pick type C1 (*NPC1*) gene transcripts were observed in infected *Ae. albopictus*, but not *Ae. aegypti*. The differences in these gene expression levels during MAYV infection could explain the variation in susceptibility observed in both mosquito species.

## 1. Introduction

Mayaro virus (MAYV) is a mosquito-borne *Alphavirus* belonging to the *Togaviridae* family, such as Chikungunya, O’nyong-nyong, Ross River, and Sindbis viruses [1,2,3]. MAYV has been discovered and isolated in 1954 from the blood of forest workers in Trinidad. More recently, several outbreaks related to MAYV have been reported in South and Central America [4], notably in Brazil, where there have been sporadic cases of Mayaro fever between 1955 and 2016 [5,6]. MAYV is also circulating in the Caribbean, in particular in Haiti, where an 8-year-old child was reported to be infected with MAYV in 2015 [7]. There have also been reports of several imported cases in France, the Netherlands, Switzerland, and Germany [8,9,10]. This situation has raised concerns about the possible expansion of this arbovirus worldwide. MAYV is classified into three major genotypes: D (dispersed), L (limited), and N (new) [11,12]. The genotype D was isolated from strains in Peru, Bolivia, Venezuela, and Trinidad, whereas genotype L was isolated in Brazil and Haiti [7], and genotype N was isolated in 2010 in Peru [12].

In humans, MAYV infection causes acute febrile illness associated with high fever, macula papular rash, chronic polyarthritis, myalgia, headache, nausea, and retro-orbital pain [3,13]. The symptomatic presentation is similar to those associated with Dengue, Chikungunya, and other acute febrile tropical diseases. The true incidence of MAYV infection is, therefore, likely to be grossly underestimated, especially in light of limited surveillance and lack of accurate diagnostic tests in much of the endemic regions. The fact that MAYV can pass undetected in areas with other ongoing arbovirus outbreaks is a cause of great concern. The documented outbreaks of Mayaro fever to date have occurred in rural communities in tropical forests [11,14]. Indeed, MAYV is primarily transmitted through the bite of tree-dwelling *Haemagogus* mosquitoes that are responsible for maintaining the sylvatic cycle [4]. Several cases of MAYV have also been recorded near cities including in urban and peri-urban residential areas in Brazil, where the domestic mosquito *Ae. aegypti* is circulating [3,15]. This observation is of major concern as it raises the possibility of the involvement of domestic mosquitoes in MAYV transmission in urban areas and may no longer be limited to rural regions. It has also been experimentally demonstrated that *Ae. aegypti* and the invasive species *Ae. albopictus* are competent vectors for MAYV [16,17,18].

Studies on the transmission of MAYV have mainly been conducted using a viral isolate belonging to the genotype D, isolated in Trinidad in 1954 [7]. The antiviral response is well documented for several arboviruses [19,20,21]; however, to the best of our knowledge, little is known about the expression of the *Aedes* mosquito’s innate immune genes in response to MAYV infection. The current study investigated the immune response in the *Ae. aegypti* and *Ae. albopictus* mosquitoes following infection with a low-passage MAYV strain belonging to the MAYV L genotype recently isolated in Haiti.

## 2. Materials and Methods

### 2.1. Virus

We used the Homo sapiens/Haiti-1/2015 MAYV strain (Genotype L; GenBank accession number KX496990) [7], a low-passaged strain isolated in 2015 from the plasma of an 8-year-old Haitian child who had a fever and abdominal pain. This strain derived from a reverse genetics system based on its GenBank. The ISA (Infectious Subgenomic Amplicons) procedure was used to implement this reverse genetics system as previously described [22,23]. Briefly, the complete viral genome flanked, respectively, at 5’ and 3’ termini by the human cytomegalovirus immediate early enhancer/promoter and the hepatitis delta ribozyme followed by the simian virus 40 polyadenylation signal was de novo synthesized in three double-stranded overlapping DNA fragments. These synthetic genes were used as a template to produce subgenomic amplicons by PCR. An equimolar mix of the three purified amplicons was used for cell transfection (HEK-293 cells, Lipofectamine 3000, Thermo Scientific, Waltham, MA, USA). Infectious cell supernatant media was then serially passaged three times in Vero-E6 cells. C6/36 mosquito cells, used for propagation of the MAYV in this study, were maintained at 28 °C in Dulbecco’s modified Eagle’s medium (DMEM; Invitrogen, Cergy Pontoise, France), supplemented with 10% fetal calf serum (FCS), as previously described [24]. Vero-E6 cells were used for viral titration and grown in DMEM, supplemented with 5% FCS (Lonza, Basel, Switzerland). Vero-E6 and C6/36 cell lines were obtained from Dr. Philippe Desprès (Pasteur Institute, Paris, France).

### 2.2. Mosquito Colonies

The *Ae*. *aegypti* Bora colony was established in 2010 in the Institut de Recherche pour le Développement (Montpellier, France), from eggs collected in French Polynesia Asian, whereas the *Ae. albopictus* colony was derived from larvae collected in La Reunion Island. The two colonies were set up to hatch under standard insectary conditions (28 ± 1 °C, 70 ± 8% RH, and 12:12 light and dark photoperiod). Larvae of both colonies were randomly seeded into plastic trays containing 1 L of tap water at a constant density of about 500 individuals per tray. Larvae were fed ad libitum with Tetramin^®^ fish food while adults were fed with a 10% sucrose solution (*w/v*).

### 2.3. Oral Infection and Dissection

Seven day-old female mosquitoes were sugar-deprived for 24 h before infectious blood-feeding. They were offered a blood meal containing 40% of washed rabbit erythrocytes from animals housed at the IRD animal facility, 5% of 100 mM ATP (Thermo Scientific), 5% human serum (Sigma, St. Louis, MO, USA), and 50% of virus in DMEM (Gibco, Thermo Scientific). The virus titer in the blood meal was adjusted to 10^6^ FFU/mL. Mosquitoes were allowed to blood feed for one hour using a Hemotek^®^ membrane feeder system with a porcine intestine membrane. After blood-feeding, only fully engorged females were kept and maintained in the same insectary condition in the bio-safety level 3 facility at IRD (Vectopôle, Montpellier, France). In parallel, female mosquitoes were engorged without virus and dissected at various time points after blood-feeding under the same experimental conditions. Mosquitoes were dissected at various time points after blood-feeding (at 3, 5, 7, and 14 dpi), and the salivary gland and midgut were transferred individually to 1.5 mL Eppendorf tubes containing 350 μL of TRK lysis buffer. The blood-feeding assays were repeated three times independently with 20 to 35 dissected mosquitoes for each species and time points (*R1*: N = 160; *R2*: N = 160; *R3*: N = 301).

### 2.4. Saliva Collection and Titration

Orally infected mosquitoes were anesthetized, wings and legs were removed, and the bodies were attached to a glass slide using double-sided tape. The proboscis was manually inserted into a 10 μL low binding pipette tip filled with 10 μL DMEM containing 2% FBS. Mosquitoes saliva was collected 30 min later in a tube and stored at −80 °C until analysis. For titration, individual mosquito saliva was homogenized in 300 μL DMEM supplemented with 2% FBS, filtered through a 0.22 μm filter, and used in a plaque assay with Vero cells, as previously described [25]. Twenty mosquitoes were analyzed for each time point (3, 5, 7, and 14 days post-oral infection).

### 2.5. Detection of MAYV in Mosquito Tissues by Real-Time PCR

Single salivary gland, midgut, or carcass was homogenized in 350 μL of TRK lysis buffer (E.Z.N.A. Total RNA kit I (OMEGA Bio-Tek, Norcross, GA, USA) using a bead Mill homogenizer (FastPrep-24, MP Biomedicals, 2 Pioneer Pl, Singapore) and total RNA was extracted according to the manufacturer’s protocol. RNA was eluted in 30 μL Diethyl pyrocarbonate-treated H_2_O. One microgram of RNA was used for reverse transcription using Moloney murine leukemia virus (M-MLV) reverse transcriptase (Promega, Charbonnieres, France), according to the manufacturer’s instructions. Maxima probe/ROX qPCR master mix (Fermentas, Saint Remy les Chevreuses, France) was used for real-time PCR. Each 25 µL reaction mixture contained 500 nM forward primer, 500 nM reverse primer, 250 nM specific probe, and 1× (final concentration) Maxima probe/ROX qPCR master mix. For MAYV, amplification in an Applied Biosystems 7300 real-time PCR system involved activation at 95 °C for 10 min followed by 40 amplification cycles of 95 °C for 15 s, 60 °C for 30 s, and 72 °C for 30 s. First, total viral RNA from the cell culture was purified using a QIAamp viral RNAkit (Qiagen, Courtaboeuf, France) following the manufacturer’s protocol. RNA standards containing RNA copies were used to construct a standard curve. A standard RT-PCR was then carried out by using primers containing the T7 promoters sequences: ((T7-MAYV_F, TAATACGACTCACTATAGGGTGCGCCTGCCAGGAGAATGCTGT and MAYV_R, TCGCCTGATGCCTTGGCCAACT) for MAYV. The PCR product was used to generate MAYV RNA fragments by in vitro transcription using a MAXIscript kit (Ambion, Austin, TX, USA). RNA was then purified by ethanol precipitation, and the RNA strands generated were determined by spectrophotometry and converted to numbers of molecular copies by using the following formula: number of *y* molecules per microliter = [(*x* grams per microliter of RNA)/(transcript length in base pairs × 340)] × 6.02 × 10²³.

### 2.6. Immune Gene Expression Analysis by Real-Time PCR

cDNA was synthesized using 0.5µg of tissues total RNA and the MMLV reverse transcription Kit, following the manufacturer’s protocol (Promega, Charbonière, France). Gene expression was quantified using real-time PCR with an Applied Biosystems 7300 real-time PCR system. RT-qPCR primers were synthesized, as shown in Table S1. Real-time PCR was performed using 2 μL of cDNA with specific primers targeting the genes of interest and 400 nM of each primer and 4 µL of Fast Eva Green Master Mix (Invitrogen; Thermo Fisher Scientific, Inc.) in an 8 µL reaction volume. The cycling conditions were 45 cycles of 95 °C for 15 s, 60 °C for 15 s, and 72 °C for 20 s. mRNA expression (fold induction) was quantified by calculating the 2^−ΔΔ*CT*^ value, with actin mRNA as an endogenous control and the mosquitoes challenged with uninfectious blood meal as control.

### 2.7. Statistical Analyses

We assessed the susceptibility of *Ae. albopictus* and *Ae. aegypti* to infection by MAYV by analyzing the rate of infection and the viral load as response variables in the midgut and salivary glands separately. To this aim, we examined the effects of three explanatory variables: mosquito “*species*” (a two-level categorical variable: *albo* and *aegypti*), “*dpi*” for day post-infection (a numerical variable), and “*replicate*” for blood-feeding assays (a three-level categorical variable: *R1*, *R2*, and *R3*). The infection rate was analyzed using a generalized linear model with a binomial error structure, and viral load was analyzed using a generalized linear model with a Gaussian error structure after log-transformation to obtain a normally distributed response variable. Maximal models included the main variables and their interactions. The significance of variables and selection of the minimal model were assessed using the ANOVA procedure within the package Car [26], which performs a type III hypothesis. The normality of residuals was checked with the Shapiro test procedure (package Stats Estimates of each three parameters were computed, and post-hoc tests (package Emmeans) were carried out to assess differences between estimates, and Bonferroni corrections were applied for multiple comparisons [27]. The comparison of the number of infectious particules and expressed as plaque-forming unit (pfu) per infected saliva was performed using the T-test. All statistical analyses were performed with R software 3.5.1 [28].

## 3. Results

### 3.1. Aedes albopictus and Aedes aegypti Infection Dynamics to MAYV

**Figure 1:**
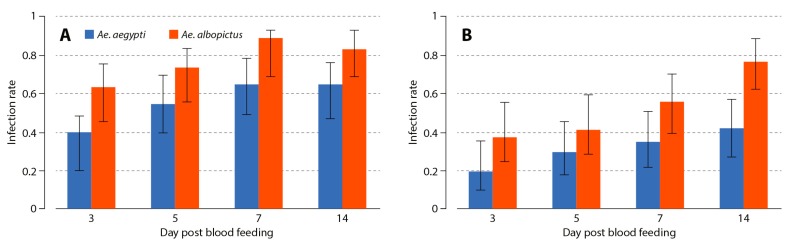
Viral infection rates after oral exposure to *Aedes aegypti* and *Aedes albopictus.* Panels (**A**,**B**) represent the rate of viral infection across time in the midgut and salivary glands, respectively. Error bars represent the 95% confidence interval.

The infection rate of MAYV was assessed in the midgut and salivary glands of the two *Aedes* species at 3, 5, 7, and 14 days following oral infection (Figure 1). Statistical analysis of the midgut infection rate showed no significant variation associated with the feeding assay (χ^2^ = 0.282, *p* = 0.868). However, midgut infection rate increased with time (χ^2^ = 9.84, *p* = 0.0017) and showed a significant difference between mosquito species (χ^2^ = 16.76, *p* < 0.001; Figure 1A): *Ae. albopictus* had a higher midgut infection rate than *Ae. aegypti* (76.6% (95% confidence interval, 0.691−0.827) vs. 53.8% (0.458–0.616], *p* < 0.001). No interaction between variables appeared significant. The proportion of infected salivary glands increased with time (χ^2^ = 15.87, *p* < 0.001) and was significantly different between mosquito species (χ^2^ = 16.78, *p* < 0.001, Figure 1B). There was no significant variation between feeding assays (χ^2^ = 0.565, *p* = 0.754) and no significant interactions. Regardless of the dpi, *Ae. albopictus* showed a higher rate of MAYV infection in salivary glands than in *Ae. aegypti* (54.3% (0.461−0.624) vs. 30.7% (0.239–0.385), *p* < 0.001).

**Figure 2:**
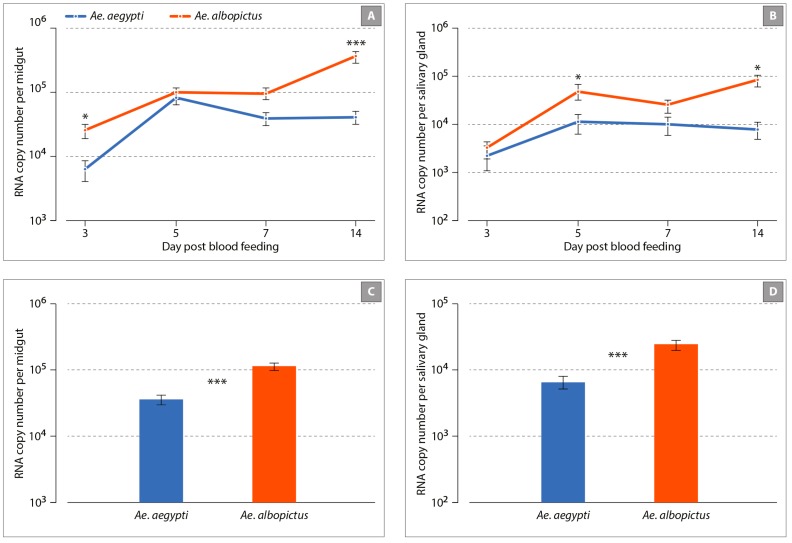
Kinetics of Mayaro (MAYV) infection of *Ae. albopictus* and *Ae. aegypti* mosquitoes. Panels (**A**,**B**) represent the number of the viral genome across time in the midgut and salivary glands, respectively. Panels (**C**,**D**) represent the mean number of viral genome for all time points. Error bars represent the standard error of the mean and asterisks indicate the significance level for statistical difference between mosquito species: * *p* < 0.05; *** *p* < 0.001; non-significant differences were not indicated for clarity.

**Figure 3:**
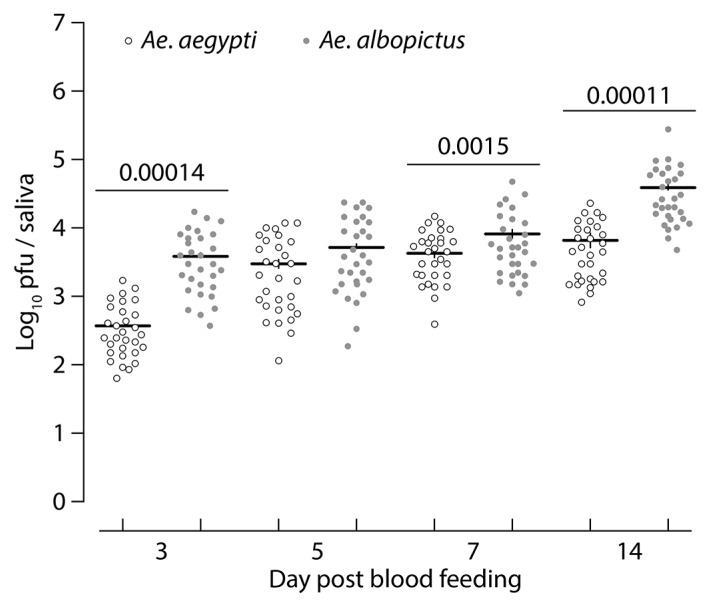
Kinetics of viral transmission after oral exposure of *Ae. albopictus* and *Ae. aegypti* to MAYV. The transmission rates were determined by titration of the saliva collected from mosquitoes at day 3, 5, 7, and 14 post-virus exposure. Each circle corresponds to the virus titer of an individual subject, and the solid horizontal line represents the mean virus titer of the group.

We next evaluated the kinetics of viral infection using RT-qPCR on the same mosquito organs and time points (Figure 2). The viral load in the midgut showed significant variation between feeding assays, alone (χ^2^ = 9.19, *p =* 0.010) or interaction with mosquito species (χ^2^ = 12.76, *p =* 0.0017) and the dpi (χ^2^ = 19.01, *p* < 0.001) (Figure 2A). Viral RNA copy numbers increased with time, and this variation was distinct in both species, as shown by the significant interaction (*species: dpi* interaction χ^2^ = 10.28, *p* = 0.0013). Overall viral load in this organ was significantly influenced by mosquito species (χ^2^ = 3.89, *p =* 0.048) with a higher titer in *Ae. albopictus* as compared to *Ae. aegypti* (RNA copy number per midgut: 1.13 × 10^5^ ± 8.7 × 10^4^ and 3.5 × 10^4^ ± 5.5 × 10^3^, respectively, *p* < 0.001) (Figure 2C). Analyzing viral load in salivary glands showed that only the three-way interaction between the feeding assays, mosquito species, and the dpi was significant (χ^2^ = 6.86, *p* = 0.032, Figure 2B). RNA copy number per salivary gland was higher in *Ae. albopictus* as compared to *Ae. aegypti*: 2.4 × 10^4^ ± 1.7 × 10^4^ and 6.5 × 10^3^ ± 4.3 × 10^3^, *p <* 0.001, Figure 2D). Mosquito saliva was collected and analyzed at 3, 5, 7, and 14 days following oral infection to determine the presence of infectious viral particles using a plaque assay. The number of infectious viral particles was significantly higher in *Ae. albopictus* as compared to *Ae. aegypti* at 3 dpi (*p* = 0.00014), 7 dpi (*p* = 0.0015), and 14 dpi (*p* = 0.00011) (Figure 3). It is also noteworthy to mention that in both species, the number of infectious viral particles increased overtime.

### 3.2. Innate Immune Response to Mayaro Virus Infection

**Figure 4:**
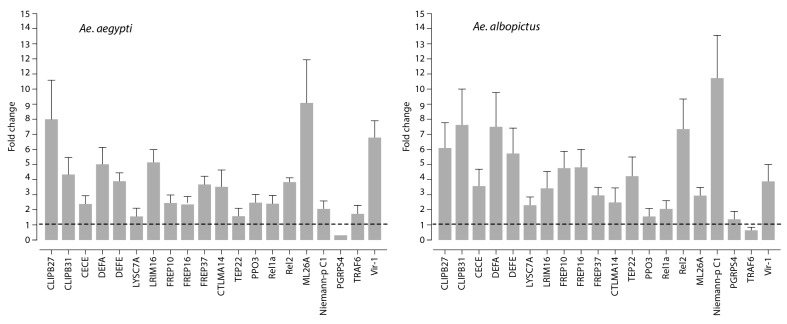
Quantification of immune gene expression. Fold change in putative immune gene expression in the midgut following the feeding of *Ae albopictus* and *Aedes aegypti* mosquitoes with MAYV-containing blood, as compared to non-infectious blood meal as a control. The gene expression profile was determined by real-time PCR at 3 days post-blood-feeding. The gene expression of mosquitoes that were fed with non-infected blood meal as a control was represented as 1 (baseline). Experiments were performed three times, and error bars represent standard error of the mean.

We analyzed the expression levels of twenty genes known to be involved in the immune response of mosquitoes in the midgut of MAYV-infected *Ae. aegypti* and *Ae. albopictus* using real-time PCR. Gene expression levels determined at an early infection stage (3 dpi) were compared with those obtained from mosquitoes challenged with a non-infectious blood meal. In both mosquito species, the analysis revealed a common expression profile of several immune genes, including CLIP-domain serine proteases (*CLIPB27* and *CLIPB31*), the anti-microbial peptides defensin A (DEFA), E (DEFE) and cecropin E (CECE), as well as the virus-inducible gene (*Vir-1*) that were significantly upregulated upon MAYV infection (Figure 4). Expression levels of the Leucine-rich repeat immune protein 16 (LRIM16), the fibrinogen-related proteins (*FREP10*, *FREP16*, and *FREP37*), and the C-type lectins mannose-binding 14 (CTLMA14) were also upregulated as a result of MAYV infection. These results notwithstanding, the transcripts of LRMI16 and FREP37 were present at a higher abundance in *Ae. aegypti*, whereas, in contrast, gene expression levels of *FREP10* and *FREP16* were higher in *Ae. albopictus*. Furthermore, MAYV infection induced the expression of *Drosophila* Relish orthologue Rel2 and Rel1a transcripts in the two mosquito species. The regulation of certain genes was found to be *Aedes* species-dependent. For instance, infection with MAYV resulted in increased gene expression levels of myeloid differentiation 2-related lipid recognition protein (*ML26A*) in the midgut of *Ae aegypti* mosquitoes, as compared to that of *Ae. albopictus* (9-fold and 3-fold induction, respectively). In contrast, virus infection significantly increased expression levels of both thio-ester containing protein 22 (TEP22) and the Niemann–Pick type C1 (*NPC1*) transcripts in *Ae. albopictus*, whereas the expression of these two genes was slightly affected in infected *Ae. aegypti* (Figure 4): In infected *Ae. albopictus* midgut, the expression of NPC1 was significantly higher (10-fold induction) as compared to the expression of the same gene in infected *Ae. aegypti* midgut (2-fold induction). In addition, the expression of several genes was either down-regulated (*PGRPS4* and *TRAF6*) or was slightly modulated (*LYSC7A* and *PP03*) at 3 days post blood-feeding with MAYV for both species.

## 4. Discussion

Since the first discovery of MAYV in Trinidad in 1954, MAYV infections have been observed in rainforest environments in which the virus was transmitted through the bite of tree-dwelling *Haemagogus* mosquitoes. However, the increase in intercontinental travel and tourism-based forest excursions has resulted in a rise in the rate of MAYV infections in urban areas [7,12]. Our study shows that urban *Ae. aegypti* and *Ae. albopictus* used in the present study were able to transmit the MAYV Haiti strain. Levels of viral RNA, however, differed between the salivary glands of these two mosquito species at 14 dpi, and numbers of infectious viral particles were lower in the saliva of *Ae. aegypti*, as compared to those in *Ae. albopictus*. The observed differences in susceptibility to infection may be attributed to genetic differences in vector competence and/or to differential mosquito immune responses against arboviruses [29,30,31,32]. Previous studies on the vector competence of *Aedes* mosquitoes to MAYV showed lower viral titers in the saliva as compared to those observed in our study [17,18]. These contradictory data could be due to the geographical origin of the mosquito and the viral strain used in the respective studies.

The present study also evaluated the early immune response in the midgut of *Ae. aegypti* and *Ae. albopictus* challenged with MAYV by feeding with a virus-containing blood meal. To this aim, expression levels of twenty immunity-related genes in these two medically important mosquito vectors were quantified. The midgut was chosen since it is the first organ encountered by the virus during a blood meal. We found that at 3 dpi, the quantity of viral RNA was higher in the midgut of MAYV-infected *Ae. albopictus*, as compared to that observed in the midgut of infected *Ae. aegypti*. Our results, furthermore, show that at this time point, MAYV infection increased transcript levels of several immune genes in both mosquito species, including the CLIP-domain serine proteases genes that have been described as immune factors in insects hemolymph [33]. These genes are involved in hemocyte-mediated immune mechanisms [34] and are known to be associated with immune pathway activation, as well as with melanization and lytic effector mechanisms [33,35,36]. Infection with MAYV induced *DEFE*, *DEFA*, and *CECE* expression that has also been reported to be upregulated upon infection of *Ae. aegypti* mosquitoes with either Dengue (DENV) or Zika (ZIKV) viruses [35,37]. These findings suggest that these genes are part of the immune response of mosquitoes to arboviruses, although their potential role in the antiviral defense of mosquitoes remains to be elucidated. An abundance of LRIM16 transcripts was also observed in MAYV-infected *Aedes* mosquitoes. The LRIM gene family plays a key role in mosquito immune response as part of the mosquito complement-like system [38,39]. This observation might suggest that the expression of this gene could be an antagonist of MAYV during midgut infection.

Our study also demonstrates that the expression level of the FREP family genes was upregulated in both *Ae. albopic*tus and *Ae. aegypti* during infection. However, *FREP10* and *FREP16* mRNA was expressed to a greater in *Ae. albopictus,* whereas the level of FREP37 transcripts was higher in *Ae. aegypti*. *FREP10* and *FREP16* mRNA expression are reportedly upregulated in DENV-infected *Ae. aegypti* [37]. During ZIKV, DENV-2, or Yellow Fever virus infection, *FREP37* transcripts were shown to be downregulated [37,40,41]. The FREP genes have been described as a family of putative pathogen recognition receptors for both *Plasmodium* and bacteria [42,43]. To the best of our knowledge, this study is the first to describe the induction of these genes during *Ae. albopictus* and *Ae. aegypti* infection with an emergent alphavirus.

The expression of another gene, *Vir1*, is also upregulated by MAYV in both mosquitoes. This gene is regulated by the Jak/STAT pathway and has also been widely implicated in mammalian immunity [44].

The immune function of the *NPC1* gene product is known to facilitate viral infection of mosquitoes and to be induced by DENV infection in both midgut and other tissue compartments [41,45]. It has been suggested that this gene is an agonist of DENV infection in *Ae. aegypti* mosquitoes [45]. Furthermore, NPC1 is a transmembrane protein that participates in cholesterol trafficking and metabolism [46,47,48]. In the present study, the increased expression of NPC1 in *Ae. albopictus* during MAYV infection suggests that this gene may prevent, or reduce, mosquito immune responses. This result could also explain the difference in susceptibility to MAYV between the two species. The *ML26A* gene has been shown to be upregulated in *Ae. aegypti* after exposure with ZIKV [37] and in a refractory strain of *Ae. aegypti* exposed to DENV [41]. This gene contains a lipid recognition domain, and its functions are associated with pathogen recognition, lipid trafficking, and metabolism [47,49,50,51]. In the present study, the abundance of *ML26A* transcripts in *Ae. aegypti,* corroborates and extends a previous report in the literature [37,45], that had not explored ML gene expression during arbovirus infection in *Ae. albopictus.* The results of our study, which shows low expression of this gene, points to the need for further investigation of the role of ML genes during arbovirus infection in *Ae. albopictus.*

In summary, we show that *Ae. aegypti* and *Ae. albopictus* display a differential immune response against MAYV, indicating differences in the molecular interactions between MAYV and these two vectors. Further studies are needed to understand the potential link between anti-viral responses and vector competence with regard to MAYV infection of *Aedes* mosquitoes.

## Supplementary Materials

The following are available online at https://www.mdpi.com/1999-4915/11/10/924/s1, Table S1: List of primers used in this study.
