# Supplementary material for: Differential Susceptibility and Innate Immune Response of Aedes aegypti and Aedes albopictus to the Haitian Strain of the Mayaro Virus
Source: Viruses. 2019 Oct 9;11(10):924. doi: 10.3390/v11100924 (PMC6832402; doi:10.3390/v11100924)
Supplement: Supplementary file 1 [file viruses-11-00924-s001.pdf]

Name	Mosquito	Sequence (5’ -3’)	Name	Mosquito	Sequence (5’ -3’)
CECE	*Ae. aegypti*	GTGATTCCCGGTCGTCTTTG	ML26A	*Ae. aegypti*	GCTACTTCAAAACGGGCGAA
		GTGAGCAAAACCAACCCGAT			GTCTTCAAGCACCTTTCGCA
	Ae .albopictus	TACCTCCGCAGCTCCTATTG		*Ae .albopictus*	CTGGTAATGGTTGGTGCGAG
		GGGTTGCCTCGTGTGTTAAG			TGTTCGTTGCCTTGTGACAG
CLIPB27	*Ae. aegypti*	AAATACGGAGGCGCAACATC	NPC1	*Ae. aegypti*	CAGCTACGTGGTCATGTTCG
		CATTTTCCAGAACACCGCCA			GGTCGTAGCCAGCTGTAGAT
	Ae .albopictus	CGCTTTAGGGATGTTTCGGG		*Ae .albopictus*	TGAATATCTTGCAGTGCGGC
		CTGAAGCGCCTCTGAAATCC			CCATCAGGCTATCGAGTGGT
CLIPB31	*Ae. aegypti*	GTCTGCTTGGCTGTGCTATC	PGRPS4	*Ae. aegypti*	TCCTACTACGGACTCCAGCT
		TGTTGCGCCTCTTCTCCTAA			GACAGCAGCGAACTTGTTCA
	Ae .albopictus	CGGTGGCTTTGGATTTCGAT		*Ae .albopictus*	TCCAGGATTCAACGATCGCT
		CCATTCACCCAAGCGAAGAG			ATTTGGGTTGAAACGAGGCC
CTLMA14	*Ae. aegypti*	GGTATTGGATCGGTGGCAAC	PPO3	*Ae. aegypti*	GCTAAGAAAGATCGCCGTGG
		AGTATCCGCGCGAAGTTCTA			AGGGTAGGCACGATTGTTCA
	Ae .albopictus	CGTGGGGAAAAGGAACACTG		*Ae .albopictus*	AGATGTTACGACGGCAATGC
		AACGATGTTCCATGCGAGTG			CCCAGCAGCCAAATCAACTT
DEFA	*Ae. aegypti*	CGCCCTTTTGCAAACTCTCT	Rel1a	*Ae. aegypti*	ACCTTCAGCAACCTGGGCAT
		ACAAGCACTATCACCAACGC			GAGATCGATGGTGGCAGGCT
	Ae .albopictus	TGACCGCCGTAATCATCGTA		*Ae .albopictus*	CGATCGTGACGGAGCAAATT
		TGTGGGTAAGCACTGGTGAT			TCCAGCAGTTCGTTCAGAGT
FREP16	*Ae. aegypti*	TTGAAAAGTGCGCAGAGGTC	Rel2	*Ae. aegypti*	TACGAGCTCCTTCAACATGC
		TTATCACTGCACAGAACGCG			AGGTCTGCAGTTGACCCTCT
	Ae .albopictus	CCACCAGTACTAGCGAACCA		*Ae .albopictus*	GTTGCGCCATAGTGTACCAG
		CTGCTGAATCTCCCATGCAC			GAAGAAGACCGCCGAATGTC
DEFE	*Ae. aegypti*	TCTAGCTCAACCACCTTGCA	TEP22	*Ae. aegypti*	GCGGGACTGATTGTACCTTT
		ATCGGTGTCAAATTCTGCGG			GAGGATATCGCCTGGTTTGT
	Ae .albopictus	CATTGGTTTCCCTCTACGCG		*Ae .albopictus*	GACAACCCAACGGAGCATTT
		ATTCCAGCCAAGTCCACTCA			CGTGAGGGCAAAGGAAGATG
FREP10	*Ae. aegypti*	TTCCTCAGGATTGGGTCGAC	TRAF6	*Ae. aegypti*	CACAAACACGTCGGAACACT
		GGCGTGTACATGATTGGCTT			TCGTCATATGCAGCGCTCTA
	Ae .albopictus	TCTGGATAGGACTCGAACGC		*Ae .albopictus*	TTGCTTGCGATCCATACTGC
		ATGAATCCCCTGCAGTTCCA			CTGCGGAACACATGTAGAGC
FREP37	*Ae. aegypti*	GGTAGTGTTGCTGGAGGACT	Vir-1	*Ae. aegypti*	GCCAAAGTCCGGTATTCTTC
		ACCTTGAGTGACATGCCGTA			TTCACGAGATCGTCAAGGTAA
	Ae .albopictus	TTTGGCAATTTGGACGGAGG		*Ae .albopictus*	TGGGCCCATCTTGATGAGTT
		ACGAATCTTCAGCAGGTCCA			CGAGCACCTGAATGACGAAG

LRIM16	*Ae. aegypti*	GCCGATGATTTTGCTGAGCT<br>AGTGATGGCAGTTTCCTCCA	Actin	*Ae. aegypti*	GAACACCCAGTCCTGCTGACA<br>TGCGTCATCTTCTCACGGTTAG
	Ae .albopictus	TTCGCGGAACACCTTTCTTG<br>CGATTCCTTGCGCCATTGTA		*Ae .albopictus*	ACGCGAAAAGTCCAAAACGA<br>CGCGGCTATCCAAAATCCAA
LYSC7	*Ae. aegypti*	TCTCCTTTTGCGGCTGAAAC<br>GAATTCCGCCCGTACAAGTC	LYSC7	*Ae .albopictus*	TGGCAGTAACTTGACCCGAT<br>CACTGCAATGACTCTCTCGC
